# Sex differences in antipsychotic efficacy and side effects in schizophrenia spectrum disorder: results from the BeSt InTro study

**DOI:** 10.1038/s41537-021-00170-3

**Published:** 2021-08-18

**Authors:** Sanne Hoekstra, Christoffer Bartz-Johannessen, Igne Sinkeviciute, Solveig K. Reitan, Rune A. Kroken, Else-Marie Løberg, Tor K. Larsen, Maria Rettenbacher, Erik Johnsen, Iris E. Sommer

**Affiliations:** 1 Department of Biomedical Sciences of Cells and Systems and Department of Psychiatry, Rijksuniversiteit Groningen (RUG), University Medical Center Groningen (UMCG), Groningen, Netherlands; 2 NORMENT - Norwegian Centre for Mental Disorders Research, Division of Psychiatry, Haukeland University Hospital, Bergen, Norway; 3 Department of Mental Health, St. Olavs Hospital, Trondheim, Norway; 4 Department of Mental Health, Faculty of Medicine and Health Sciences, Norwegian University of Science and Technology (NTNU), Trondheim, Norway; 5 Department of Clinical Medicine, University of Bergen, Bergen, Norway; 6 Department of Addiction Medicine, Division of Psychiatry, Haukeland University Hospital, Bergen, Norway; 7 Division of Psychiatry, Haukeland University Hospital, Bergen, Norway; 8 Department of Clinical Psychology, University of Bergen, Bergen, Norway; 9 Stavanger University Hospital, Regional Centre for Clinical Research in Psychosis, TIPS, Bergen, Norway; 10 Department of Psychiatry, Medical University of Innsbruck, Innsbruck, Austria

**Keywords:** Schizophrenia, Pharmacology

## Abstract

Current guidelines for patients with schizophrenia spectrum disease do not take sex differences into account, which may result in inappropriate sex-specific treatment. In the BeSt InTro study, a total of 144 patients (93 men and 51 women) with a schizophrenia spectrum diagnosis and ongoing psychosis were included and randomized to amisulpride, aripiprazole, or olanzapine in flexible dose. This trial is registered with ClinicalTrials.gov (NCT01446328). Primary outcomes were sex differences in dose, dose-corrected serum levels, efficacy, and tolerability. Dosing was higher for men than for women in the aripiprazole group (*p* = 0.025) and, at trend level, in the olanzapine group (*p* = 0.056). Dose-corrected serum levels were 71.9% higher in women than in men for amisulpride (*p* = 0.019) and 55.8% higher in women than in men for aripiprazole (*p* = 0.049). In the amisulpride group, men had a faster decrease in psychotic symptoms than women (*p* = 0.003). Moreover, amisulpride was more effective than the other medications in men but not in women. Prolactin levels were higher in women than in men, especially for amisulpride (*p* < 0.001). Also, women had higher BMI increase on amisulpride compared to the two other antipsychotics (*p* < 0.001). We conclude that clinicians should be aware of the risks of overdosing in women, especially for amisulpride and aripiprazole. Amisulpride is highly effective in men, but in women, amisulpride showed more severe side effects and may thus not be the drug of first choice. Our study shows that sex differences should be taken into account in future studies on antipsychotics. Future research is warranted to evaluate these preliminary results.

## Introduction

Sex differences in the epidemiology and course of primary psychotic disorder have been reported consistently, with a slightly higher life-time risk in men and a higher age of onset in women. Findings regarding symptomatology in these patients are less conclusive. However, it is often reported that men have more negative symptoms, while women have more affective symptoms. Women, especially at premenopausal age, are often stated to have a better course of illness than men^1^, which might be explained by protective effects of estrogen, low rates of comorbid abuse, and better treatment response^2,3^. Individualized approaches become increasingly important in psychiatry and attention is paid to sex differences in antipsychotic treatment response. A recent compilation study with 506 samples for amisulpride, 1610 samples for aripiprazole, and 10,268 samples for olanzapine found higher antipsychotic concentration–dose ratios in women than in men^4^. Higher serum levels in women could be explained by sex differences in the pharmacokinetics of antipsychotic drugs^3^. Gastric emptying and gastrointestinal transit time are slower in women, which promotes absorption^5,6^. In addition, women have a larger volume of distribution for antipsychotics, which could increase the half-life of the drugs^7^ Moreover, gastrointestinal and renal elimination routes are slower in women than in men^3^. Regarding treatment response, higher efficacy in women compared to that in men was previously reported, but results are inconsistent and the influence of sex is not the same for all drugs^8^. As for side effects, Seeman^3,9^ concluded that women experience more side effects than men and that, for example, weight gain may be more distressing for women. Also, hyperprolactinemia was found more frequent and occurred at lower doses in women than in men^10^.

Current guidelines do not take sex differences into account and recommend the same antipsychotic drug doses for men and women, but this might not be the most appropriate treatment strategy^3^. However, similar dosing for both sexes conforms to the British guideline from the National Institute of Health and Clinical Excellence^11^ and the American guideline from The Schizophrenia Patients Outcomes Research Team^12^. Also, sex is not mentioned in The Summary of Product Characteristics of amisulpride and aripiprazole. For olanzapine, a lower starting dose should be considered when one of the following factors in present: female sex, geriatric age, non-smoking status^13^. Future research is warranted since data on sex-specific treatment, including sex-specific dosing, blood levels, efficacy, and side effects of different antipsychotics, is still inconclusive. In the current study, we evaluated sex differences in dose, dose-corrected serum levels, efficacy, and tolerability. For this purpose, we used data from the BeSt InTro project in which amisulpride, aripiprazole, and olanzapine were compared in a head-to-head trial. The primary outcome of the BeSt InTro study was change in the Positive and Negative Syndrome Scale (PANSS) after 52 weeks of treatment.

## Results

### Baseline demographic and clinical characteristics

A total of 144 patients (93 men and 51 women) were enrolled; 52 patients used amisulpride (25 men and 27 women), 51 used aripiprazole (39 men and 12 women), and 41 used olanzapine (29 men and 12 women). Baseline characteristics for both sexes are presented in Table 1. At baseline, no significant sex differences in symptom severity and side effects were found.

**Table 1:** Baseline mean (SD) demographic and clinical characteristics by sex.

	Men (*n* = 93)	Women (*n* = 51)	*p* value	Effect size
Age	30.5 (11.6)	33.8 (14.4)	0.157	0.261
Diagnosis: schizophrenia	54/93 (58%)	30/51 (59%)	1.000	0.015
Diagnosis: schizotypal	2/93 (2%)	0/51 (0%)	0.539	0.294
Diagnosis: Delusional disorder	13/93 (14%)	8/51 (16%)	0.975	0.048
Diagnosis: Brief psychotic disorder	14/93 (15%)	4/51 (8%)	0.323	0.229
Diagnosis: Schizo-affective	2/93 (2%)	8/51 (16%)	0.004*	0.520
Diagnosis: Other	0/93 (0%)	1/51 (2%)	0.354	0.281
Diagnosis: Unspecified	8/93 (9%)	0/51 (0%)	**0.051**	0.595
Antipsychotic-naive	36/93 (39%)	20/51 (39%)	1.000	0.055
Smoking	56/93 (60%)	29/51 (57%)	0.753	0.068
Abuse/dependence—alcohol	10/93 (11%)	3/51 (6%)	0.374	0.178
Abuse/dependence—drugs	24/93 (26%)	3/51 (6%)	0.003*	0.578
PANSS total	78.1 (15.6)	79.1 (16.6)	0.704	0.063
PANSS positive	21.2 (4.90)	21.3 (4.60)	0.844	0.021
PANSS negative	17.8 (5.80)	17.8 (6.70)	0.992	0.000
PANSS general	39.1 (8.90)	40.0 (8.10)	0.535	0.104
Neurologic side effects	0.70 (0.60)	0.50 (0.50)	0.183	0.353
Sexual side effects	0.48 (0.42)	0.33 (0.61)	0.177	0.303
BMI (kg/m^2^)	25.1 (4.70)	26.5 (7.80)	0.268	0.234
Glucose (mmol/L)	5.40 (1.40)	5.20 (0.90)	0.303	0.160
Prolactin (mIU/L)	290.3 (195.9)	467.8 (499.2)	**0.021**	0.529

Data are *n* (%) or mean (SD). Differences significant with *α* = 0.05 are shown in bold. *p* Values with asterisk (*) remained significant after FDR correction. Cohen’s *d* was used for mean values and Cohen’s *h* was used for proportions.

*n* number in the sample, *antipsychotic-naive* no previous exposure to antipsychotic drugs, *PANSS* Positive and Negative Syndrome Scale, *BMI* body mass index.

### Drug doses

Mean defined daily doses (DDDs) over the follow-up period (SD) were similar for men and women in the amisulpride group; 0.98 (0.54) for men and 0.97 (0.61) for women (*p* = 0.945). For aripiprazole, the mean DDD of 1.10 (0.60) for men was significantly higher than the mean DDD of 0.86 (0.49) for women (*p* = 0.025). For olanzapine, the difference was trend-wise significant, with a mean DDD of 1.28 (0.77) for men and 1.11 (0.24) for women (*p* = 0.056).

### Serum blood levels

For amisulpride and aripiprazole, similar dosing resulted in significantly higher serum levels in women than in men. The serum levels, corresponding to DDD = 1, were 458.1 (307.4) nmol/L for men and 787.7 (544.2) nmol/L for women in the amisulpride group (*p* = 0.019). Serum levels in the aripiprazole group were 471.9 (373.4) nmol/L for men and 735.0 (456.2) nmol/L for women (*p* = 0.049). In the olanzapine group, serum blood levels were 78.7 (57.5) nmol/L for men and 84.8 (44.3) nmol/L for women (*p* = 0.879).

### PANSS score—comparison between men and women

**Fig. 1:**
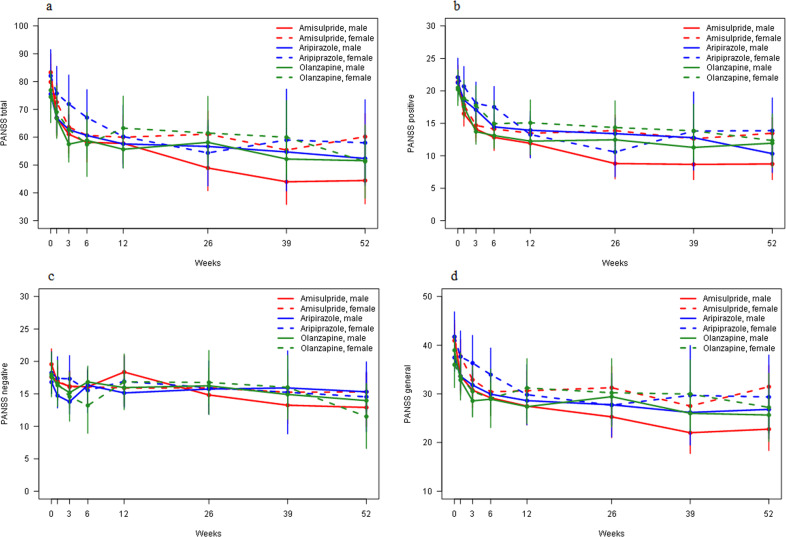
PANSS score reduction per timepoint. This figure shows the PANSS score reduction for PANSS total (**a**), PANSS positive (**b**), PANSS negative (**c**), and PANSS general (**d**) at different timepoints (0, 1, 3, 6, 12, 26, 39, and 52 weeks). All antipsychotic medications were analyzed for men and women separately. Sample sizes were as follows: amisulpride male group *n* = 25; amisulpride female group *n* = 27; aripiprazole male group *n* = 39; aripiprazole female group *n* = 12; olanzapine male group *n* = 29; olanzapine female group *n* = 12. The analysis is based on a linear mixed model with time as a categorical variable. The error bars show the standard errors of the PANSS estimates at each timepoint. *PANSS* Positive and Negative Symptom Scale.

In the amisulpride group, men showed larger reduction in overall symptom severity than women. The mean weekly reduction in PANSS total score was 0.59 (0.07) points in men and 0.30 (0.07) points in women (*p* = 0.003). A post hoc analysis demonstrated that men had significantly better improvement of PANSS total score than women at 26 (*p* = 0.009), 39 (*p* = 0.010), and 52 weeks (*p* = 0.002). No difference in overall symptom reduction was observed for men and women in the aripiprazole group (*p* = 0.265) nor in the olanzapine group (*p* = 0.776). Results were similar for all PANSS subscales (Table 2, Fig. 1, and Supplementary Table 1).

**Table 2:** Comparison between men and women for PANSS score reduction.

	Amisulpride (*n* = 52)			Aripiprazole (*n* = 51)			Olanzapine (*n* = 41)		
	Men (*n* = 25)	Women (*n* = 27)	*p* value	Men (*n* = 39)	Women (*n* = 12)	*p* value	Men (*n* = 29)	Women (*n* = 12)	*p* value
PANSS total	−0.59 (0.07)	−0.30 (0.07)	0.003*	−0.37 (0.07)	−0.53 (0.13)	0.265	−0.33 (0.06)	−0.30 (0.11)	0.776
PANSS positive	−0.20 (0.02)	−0.11 (0.02)	0.004*	−0.18 (0.02)	−0.21 (0.04)	0.507	−0.12 (0.02)	−0.13 (0.04)	0.789
PANSS negative	−0.10 (0.02)	−0.03 (0.02)	**0.028**	−0.00 (0.02)	−0.06 (0.04)	0.197	−0.05 (0.02)	−0.06 (0.04)	0.812
PANSS general	−0.29 (0.04)	−0.16 (0.04)	**0.014**	−0.19 (0.04)	−0.26 (0.07)	0.360	−0.16 (0.03)	−0.11 (0.06)	0.431

Analysis based on linear mixed model with time as continuous variable. PANSS score decrease per week is presented with SE. Differences significant with *α* = 0.05 are shown in bold. *p* Values with asterisk (*) remained significant after FDR correction.

*n* number in the total sample, *PANSS* Positive and Negative Syndrome Scale.

### PANSS score—comparison between amisulpride, aripiprazole, and olanzapine in men

The effect of time on PANSS total score decrease differed between amisulpride and aripiprazole (*p* = 0.014) and between amisulpride and olanzapine (*p* = 0.004), with a weekly reduction of 0.60 (0.07) points in the amisulpride group, 0.35 (0.07) points in the aripiprazole group and 0.33 (0.06) points in the olanzapine group. In the post hoc analysis, amisulpride was superior to aripiprazole at 3 (*p* = 0.031), 6 (*p* = 0.025), 26 (*p* = 0.003), 39 (*p* = 0.0007), and 52 weeks (*p* = 0.008). Amisulpride was superior to olanzapine at 26 (*p* = 0.005), 39 (*p* = 0.008), and 52 weeks (*p* = 0.013) Results for the PANSS subscales were largely similar (Fig. 1 and Supplementary Tables 1 and 2).

### PANSS score—comparison between amisulpride, aripiprazole, and olanzapine in women

The effect of time on PANSS total score decrease did not differ between amisulpride and aripiprazole (*p* = 0.118) nor between amisulpride and olanzapine (*p* = 0.956). The only difference in effect of time on PANSS score was between amisulpride and aripiprazole for the PANSS positive subscale; women in the aripiprazole group had greater weekly reduction than women in the amisulpride group, but this was not confirmed at any timepoint in the post hoc analysis (Fig. 1 and Supplementary Tables 1 and 2).

### Side effects—comparison between men and women

Dystonia, rigidity, hypokinesia/akinesia, hyperkinesia, tremor, akathisia, epileptic seizures, and paresthesia were included as neurologic side effects and the average Udvalg for Kliniske Undersøgelser side effect score (UKU score) on these items over all timepoints was used. The mean UKU score for neurologic symptoms did not differ between men and women for amisulpride (*p* = 0.376), aripiprazole (*p* = 0.699), and olanzapine (*p* = 0.643). Symptoms included in the category of sexual side effects were increased sexual desire, diminished sexual desire, erectile dysfunction, ejaculatory dysfunction, orgasmic dysfunction, and dry vagina during intercourse. Again, the average score on these items over all timepoints was used. Statistical significance was almost reached in the amisulpride and olanzapine group, with a mean UKU score of 0.37 (0.48) in women and 0.26 (0.42) in men (*p* = 0.051) in the amisulpride group and a mean score of 0.34 (0.54) in women compared to 0.18 (0.35) in men in the olanzapine group (*p* = 0.059). Mean body mass index (BMI) increase and mean glucose levels were included as metabolic side effects. Men had more increase in BMI than women on aripiprazole, with a mean increase of 0.64 (1.28) kg/m^2^ in men and a mean decrease of 0.04 (1.46) kg/m^2^ in women (*p* = 0.016), and on olanzapine, with a mean BMI increase of 1.48 (1.27) kg/m^2^ in men, compared to a mean increase of 0.24 (1.25) kg/m^2^ in women (*p* < 0.001). For glucose levels, the only significant difference was in the olanzapine group; men had a mean glucose level of 5.40 (1.76) mmol/L throughout the study, which was significantly higher than the mean level of 5.00 (0.68) mmol/L in women (*p* = 0.032). Lastly, the mean prolactin level of 1869.2 (1212.5) mIU/L for women in the amisulpride group was more than twice as high as the mean prolactin level of 920.3 (553.0) mIU/L in men (*p* < 0.001). In the olanzapine group, women had a mean prolactin level of 667.7 (542.1) mIU/L, which was also significantly higher than the prolactin level of 376.5 (223.5) mIU/L in men (*p* = 0.001) (see Table 3).

**Table 3:** Comparison between men and women for mean side effects.

	Amisulpride (*n* = 52)			
	Men (*n* = 25)	Women (*n* = 27)	*p* value	Effect size
Neurologic side effects (UKU)	0.37 (0.56)	0.31 (0.38)	0.376	0.126
Sexual side effects (UKU)	0.26 (0.42)	0.37 (0.48)	0.051	0.243
BMI increase (kg/m^2^)	0.90 (1.14)	1.34 (2.27)	0.111	0.242
Glucose level (mmol/L)	5.08 (0.62)	5.15 (0.82)	0.426	0.096
Prolactin level (mIU/L)	920.3 (553.0)	1869.2 (1212.5)	<0.001*	1.013

	Aripiprazole (*n* = 51)			
	Men (*n* = 39)	Women (*n* = 12)	*p* value	Effect size
Neurologic side effects (UKU)	0.46 (0.44)	0.49 (0.40)	0.699	0.070
Sexual side effects (UKU)	0.34 (0.43)	0.23 (0.44)	0.114	0.255
BMI increase (kg/m^2^)	0.64 (1.28)	−0.04 (1.46)	**0.016**	0.454
Glucose level (mmol/L)	5.71 (1.45)	5.47 (1.63)	0.366	0.161
Prolactin level (mIU/L)	265.9 (328.4)	307.4 (162.3)	0.216	0.139

	Olanzapine (*n* = 41)			
	Men (*n* = 29)	Women (*n* = 12)	*p* value	Effect size
Neurologic side effects (UKU)	0.34 (0.47)	0.38 (0.46)	0.643	0.086
Sexual side effects (UKU)	0.18 (0.35)	0.34 (0.54)	0.059	0.388
BMI increase (kg/m^2^)	1.48 (1.27)	0.24 (1.25)	<0.001*	0.981
Glucose level (mmol/L)	5.40 (1.76)	5.00 (0.68)	**0.032**	0.026
Prolactin level (mIU/L)	376.6 (223.5)	667.7 (542.1)	0.001*	0.845

Data are mean values over all timepoints of the follow-up period with SD. Differences significant with *α* = 0.05 are shown in bold. *p* Values with asterisk (*) remained significant after FDR correction. Cohen’s *d* was used to calculate effect sizes.

*n* number in the total sample, *BMI* body mass index.

### Side effects—comparison between amisulpride, aripiprazole, and olanzapine in men

No difference was observed between the three types of medication for neurologic side effects (*p* = 0.155). Regarding sexual side effects, a significant difference was found between the groups (*p* = 0.006) and a post hoc analysis revealed the mean UKU score of 0.34 (0.43) in the aripiprazole group to be higher than the score of 0.18 (0.35) in the olanzapine group (*p* = 0.004). Looking at metabolic side effects, a significant difference in both BMI increase (*p* < 0.001) and glucose levels (*p* = 0.001) was observed. In a post hoc analysis, this was explained by a higher mean BMI increase of 1.48 (1.27) kg/m^2^ in the olanzapine group compared to an increase of 0.90 (1.14) kg/m^2^ in the amisulpride group (*p* = 0.014) and 0.64 (1.28) kg/m^2^ in the aripiprazole group (*p* < 0.001), and a mean glucose level of 5.71 (1.45) mmol/L in the aripiprazole group compared to 5.08 (0.62) mmol/L in the amisulpride group (*p* < 0.001). Prolactin levels were different between medications (*p* < 0.001); the mean level of 920.3 (553.0) mIU/L for amisulpride was significantly higher than the level of 265.9 (328.4) mIU/L for aripiprazole (*p* < 0.001) and 376.5 (223.5) mIU/L for olanzapine (*p* < 0.001). The prolactin level in the olanzapine group was also significantly higher than the prolactin level in the aripiprazole group (*p* = 0.031) (Supplementary Tables 3 and 4).

### Side effects—comparison between amisulpride, aripiprazole, and olanzapine in women

A significant difference between medications was found (*p* = 0.033) for neurologic side effects and a post hoc analysis showed this was due to the higher mean UKU score of 0.49 (0.40) in the aripiprazole group compared to 0.31 (0.38) in the amisulpride group (*p* = 0.025). No significant difference was found between the medications for sexual side effects (*p* = 0.183). Comparison of the three medication groups regarding BMI increase demonstrated a significant difference (*p* < 0.001). More specifically, a mean increase of 1.34 (2.27) kg/m^2^ in the amisulpride group was found, which was higher than the decrease of 0.04 (1.46) kg/m^2^ in the aripiprazole group (*p* < 0.001) and the increase of 0.24 (1.25) kg/m^2^ in the olanzapine group (p = 0.041). No differences were found between medications for glucose level (*p* = 0.082). Prolactin levels were different in the medication groups (*p* < 0.001). Women in the amisulpride group had a mean prolactin level of 1869.2 (1212.5) mIU/L, which was much higher than the mean level of 307.4 (162.3) mIU/L in the aripiprazole group (*p* < 0.001) and 667.7 (542.1) mIU/L in the olanzapine group (*p* < 0.001). No difference between medications was observed for mean UKU score on tension in breasts (*p* = 0.078). However, the UKU score on decreased menstruation differed between groups (*p* < 0.001). More specifically, the mean score of 0.65 (1.15) in the amisulpride group was higher compared to a score of 0.20 (0.66) in the aripiprazole group (*p* = 0.017) and 0.00 (0.00) in the olanzapine group (*p* < 0.001) (Supplementary Tables 3 and 4).

## Discussion

The primary outcome of the BeSt InTro study was change in PANSS total score over 52 weeks, which was highest for patients in the amisulpride group, compared to the aripiprazole and olanzapine groups. The aim of the current study was to evaluate sex differences in dosing, dose-corrected serum blood levels, efficacy, and side effects experienced with amisulpride, aripiprazole, and olanzapine. The naturalistic design provided men with similar doses as women for amisulpride, whereas for aripiprazole and olanzapine, men received higher doses. Dose-corrected serum levels were significantly higher in women for amisulpride and aripiprazole, but not for olanzapine. Men had better treatment response to amisulpride than women, as measured by PANSS score. Amisulpride was significantly more effective than the other medication types for men, but not for women. Side effects were generally mild. However, prolactin levels were significantly higher in women, especially for amisulpride. Also, BMI increase was highest for men on olanzapine, while women had highest BMI increase on amisulpride. These findings may indicate that amisulpride should be the drug of first choice for men with an acute psychotic episode, but not for women. Clinicians were free to titrate dose based on efficacy and side effects. Based on clinical evaluation, clinicians used similar dosing for men and women with regard to amisulpride, but men received higher mean doses than women for aripiprazole and olanzapine. Although similar dosing would conform to current guidelines, our study indicates that this may result in dissimilar blood serum levels for amisulpride and aripiprazole. Future research is warranted to evaluate sex-specific dosing. Amisulpride and aripiprazole, but not olanzapine, showed significantly higher serum levels, corrected for daily dose, in women than in men. Differences were in the range of an 72% increase for women in the amisulpride group and a 56% increase for women in the aripiprazole group. In addition to aforementioned sex differences in absorption, distribution, and elimination, sex differences in metabolizing enzymes of the P450 (CYP450) system could influence serum levels as well^14^. Amisulpride is mostly eliminated by renal clearance as an unchanged substance. A lower glomerular filtration rate in women probably explains the higher serum levels for amisulpride^15^. Aripiprazole is metabolized by CYP3A4 and CYP2D6, and it has been suggested that women have higher CYP3A4 activity than men, which would contradict higher serum levels in women^16^. However, interpretation remains difficult, due to inconsistent results and wide variation in CYP3A4 activity^17^. Our observation of higher dose-corrected serum levels in women than in men for aripiprazole may rather be a strong indication of longer half-life in women. Olanzapine is mainly metabolized by CYP1A2. Women have lower CYP1A2 activity, which could explain higher serum levels in women^18^. In the current study, we could not replicate this finding. However, sex is not the only influencing factor on CYP1A2 activity, as smoking was found to influence CYP1A2 function as well. Smoking is often more prevalent in men and could contribute to sex differences, as tobacco induces CYP1A2, reducing serum levels^19^. In our sample, smoking was almost as common among women (57%) as among men (60%), which may explain similar blood levels in these groups. In the amisulpride group, men had better symptom reduction than women, especially at the end of follow-up. After 52 weeks, men in the amisulpride group had a reduction of 73.0% of the baseline PANSS total score, while women had a reduction of 39.5%. This is a remarkable finding, as amisulpride serum blood levels were lower in males. The 73.0% reduction in men corresponds to the category very much improved on the Clinical Global Impression scale. The 39.5% reduction found in women falls between the categories minimally improved and much improved^20^. These findings are not in line with the findings of previous studies. In the EUFEST study^8^, no difference in PANSS score reduction between men and women in the amisulpride group was found. Moreover, in the OPTiMiSE study^21^, remission rates after 4 weeks of amisulpride flexible dose were similar in both sexes (30% in men vs 24% in women, *p* = 0.20). In both the EUFEST and the OPTiMiSE study, patients had a first-episode psychosis and were mostly drug naive, while in the current study only around 40% of the patients were drug naive. We did not find any differences in efficacy between men and women in the aripiprazole and olanzapine group. To our knowledge, no previous studies investigated sex differences in efficacy of aripiprazole. Regarding olanzapine, Goldstein et al.^22^ reported a greater improvement in severity of psychotic symptoms in women than in men. The EUFEST study yielded similar results. However, better treatment response and prognostic outcome for women could be questioned, as, in line with our study, the SOHO study^23^ did not find a sex difference in clinical efficacy of olanzapine. It is often stated that women have a better illness course due to fewer and shorter hospitalizations than men, but findings are inconsistent^1^. In a nationwide cohort study, women with schizophrenia spectrum disorder did not have fewer admissions for psychosis or shorter hospital stays than men^2^. In line with this finding, a meta-analysis found largely similar recovery rates for women (12.9%) and men (12.1%)^24^. When comparing the three medication types for both sexes separately, we found that amisulpride is more effective than aripiprazole and olanzapine in men, but not in women. As amisulpride did not produce more side effects in men, this drug can be recommended as the first-choice antipsychotic for men. In a substudy of the EUFEST project^25^, use of amisulpride was also found to be the strongest predictor of clinical response and remission. Based on a balance between efficacy and side effects, amisulpride may not be our first choice for women since it does not provide higher efficacy than the other medications and side effects are higher. In our study, sex differences were found for BMI increase and prolactin levels. Men had highest BMI increase on olanzapine, while women had highest BMI increase on amisulpride. This could be related to the very high amisulpride serum blood levels in women. In a subanalysis of the EUFEST study, olanzapine also caused most weight gain after 52 weeks, but it was followed by amisulpride with a non-significant difference^26^. BMI increase during treatment with amisulpride is not supported by all literature^27^. However, this study did not compare male and female patients. Our findings indicate that BMI increase may be sex-specific and that the drug that causes most weight gain in men is not necessarily the drug that causes most weight gain in women. Both men and women had highest prolactin levels on amisulpride, and the reference ranges of <424 mUI/L for men and <530 mUI/L for women^28^ were clearly exceeded. These findings are in line with a recent comprehensive review that stated amisulpride is the antipsychotic with the potential for maximum prolactin elevation^29^. In our sample, mean prolactin levels in women on amisulpride were 103.1% higher than mean prolactin levels in men on amisulpride. Prolactin levels were already higher in women at baseline, but increase in women was still considerably more pronounced than increase in men, which is in line with recent literature^28^. Hyperprolactinemia in women may lead to hypoestrogenism, which can cause gynecomastia and amenorrhea. In the current study, women in the amisulpride group reported more amenorrhea, whereas they did not report more gynecomastia than women in the other medication groups. Of note, hypoestrogenism could have negatively affected the course of illness in women in our study, as estrogens have a protective effect in psychosis^30^. This study has several limitations, and our results are subject to the limitations related to the original study design. These study limitations are described elsewhere^31^. The BeSt InTro study was powered and aimed at measuring efficacy and did not primarily focus on sex differences. As in most randomized controlled trials, males were overrepresented, which decreased the power to detect sex differences. The relatively low number of participants in both sex groups, but especially women, increased the probability of type II errors. We decided to use per protocol (PP) analyses only in the current study, due to a high percentage of women switching in randomization groups. This might have led to selection bias. However, we could not detect any major differences between our intention to treat (ITT) and PP groups. The sex differences we found in the amisulpride group followed a similar pattern in both analyses. Our study also has important strengths. All participants were assessed for efficacy and side effects at many timepoints. Dosing and serum levels were measured at each timepoint together with prolactin levels, which provides a wealth of data and a follow-up time longer than most studies. In conclusion, we found that similar dosing between men and women for amisulpride leads to 71.9% higher drug serum levels and 103.1% higher prolactin levels in women. Despite higher amisulpride blood levels in women, recovery of symptoms was better in men. Based on efficacy, amisulpride is probably the antipsychotic medication of first choice in men, but not in women. In women, efficacy of amisulpride was not superior to that of aripiprazole or olanzapine, while prolactin rise and BMI increase were maximal on this drug. Based on our data, aripiprazole may be a good alternative for women, as it does not cause BMI or prolactin increase, while efficacy is similar to that of the other two drugs. Future research including a larger sample size is warranted to validate these findings. Our study shows that sex should be taken into account in future research and there may be a need for separate guidelines and dosing schedules for women and men. A first step in future research would be to determine optimal dosing for women for commonly used antipsychotic drugs.

## Methods

### Study design and participants

The design and randomization procedure of the BeSt InTro study were published in more detail elsewhere^31^. The BeSt InTro project was a prospective, pragmatic, randomized, rater-blind, longitudinal study with a 52 week follow-up in which amisulpride, aripiprazole, and olanzapine were compared. Patients aged ≥18 years with a diagnosis within the schizophrenia spectrum according to the International Classification of Diseases-10 diagnoses F20–F29 and with an indication for oral antipsychotic drug therapy were included. Patients were eligible for the study if they had a score of ≥4 on at least one of the following items in the PANSS: delusions, hallucinatory behavior, grandiosity, suspiciousness/persecution, or unusual thought content. All patients provided written informed consent before inclusion. Study visits were at baseline, 1, 3, 6, 12, 26, 39, and 52 weeks. This trial is registered with ClinicalTrials.gov, number NCT01446328.

### Randomization procedure

Participants were randomized to a sequence of the three antipsychotics and preferably the first drug on the list was chosen as the study drug. If the first drug could not be used due to contra-indications or prior negative experiences, the next drug on the list was offered. Randomization was open to the treating psychiatrist, the physician, and the patient, but all research personnel involved in the study assessments was blinded. Decisions concerning further dosing, concomitant use of other drugs, and antipsychotic drug switches were made by the treating psychiatrist. Randomization groups formed the basis for ITT analyses, whereas the drug that was actually chosen formed the basis for PP analyses. In the current study, 24 (16.7%) of the patients did not choose the first drug in their sequence. For men, there were no statistically significant differences in switching among the randomization groups (Fischer’s exact test: *p* = 0.155) meaning it can be assumed as mostly random. For women, the difference in switching among the randomization groups was close to significance (Fischer’s exact test: *p* = 0.058), as 8/19 (42.1%) of the women who were randomized to olanzapine actually took amisulpride. In order to better capture the actual drug effects, we decided to use PP groups instead of ITT groups in the current study.

### Assessment

Doses were analyzed as DDDs, with 1 DDD equaling 400 mg for amisulpride, 15 mg for aripiprazole, and 10 mg for olanzapine (World Health Organization^32^). Symptom change was assessed with the structured clinical interview for the PANSS^33^ and the patient-rated version of the UKU side effect rating scale was used to follow the development of side effects, with 0 = no side effects, 1 = mild side effects that do not interfere with performance, 2 = side effects that interfere moderately with performance and 3 = side effects that interfere markedly with performance^34^.

### Statistical analysis

Statistical analyses were performed using R. Shapiro–Wilk test was performed to test for normality of the data. All variables were normally distributed, except for prolactin and glucose. Analyses using log-transformation were performed but did not change our findings. Baseline characteristics of men and women were compared with two-tailed *t* tests, chi-square tests, and Fisher’s exact tests, as appropriate. A *p* value < 0.05 was taken as the level of statistical significance. Mean doses over all timepoints of the follow-up period were analyzed with two-tailed *t* tests and a simple linear regression model was used to analyze blood serum levels, including DDD as a covariate in the model to correct for dose. For PANSS score decrease, a linear mixed model was used with PANSS score as the dependent variable. Sex, time, and their interaction were included as fixed effects and a random intercept for each individual was included as a random effect. Each medication group was analyzed separately. Time was included as a continuous fixed variable first with mean weekly PANSS score change as outcome, to limit the number of comparisons. If a significant difference was found, post hoc analyses were performed with time as a categorical fixed variable to look for sex differences at specific timepoints. The same procedure was followed to compare medications for each sex, only medication type was included in the model instead of sex, and amisulpride was chosen as reference category. For side effects, mean values or levels over all timepoints of the follow-up period were compared between men and women with a two-tailed *t* test and between the three medications with ANOVA. When significant, Tukey’s post hoc analyses were performed. False discovery rate correction according to the Benjamini and Hochberg method was used for multiple testing. Cohen’s *d* was used to calculate effect sizes for continuous variables, whereas Cohen’s *h* was used to calculate effects sizes for discrete variables.

### Ethical approval

The BeSt InTro study was approved in Norway by the Regional Committee for Medical Research Ethics and the Norwegian Medicines Agency. In Austria, the project was approved by the Ethical Committee for the Medical University of Innsbruck and the Austrian Federal Office for Safety in Health Care.

### Reporting summary

Further information on research design is available in the Nature Research Reporting Summary linked to this article.

## Supplementary information

Supplementary Information

Reporting Summary

## Data Availability

According to the Norwegian law, data sharing requires approvals from the Regional Committees for Medical and Health Research Ethics and from the Data Protection Officer at Haukeland University Hospital. The data are therefore not publicly available. The data that support these findings can be provided by E.J., Norwegian Centre for Mental Disorders research at Haukeland University Hospital, upon reasonable request.

## Code availability

All analyses were conducted in R and the codes used are provided in the Supplementary Information files.

## References

[1] Riecher-Rössler A, Butler S, Kulkarni J (2018). Sex and gender differences in schizophrenic psychoses—a critical review. Arch. Women’s. Ment. Health.

[2] Sommer IE, Tiihonen J, van Mourik A, Tanskanen A, Taipale H (2020). The clinical course of schizophrenia in women and men—a nation-wide cohort study. npj Schizophr..

[3] Seeman, M. V. Men and women respond differently to antipsychotic drugs. *Neuropharmacology* **163**, 107631 (2020). 10.1016/j.neuropharm.2019.05.008 31077728

[4] Jönsson AK, Spigset O, Reis M (2019). A compilation of serum concentrations of 12 antipsychotic drugs in a therapeutic drug monitoring setting. Ther. Drug Monit..

[5] Bennink R (1998). Comparison of total and compartmental gastric emptying and antral motility between healthy men and women. Eur. J. Nucl. Med..

[6] Soldin, O. P., Chung, S. H. & Mattison, D. R. Sex differences in drug disposition. *J. Biomed. Biotechnol.* **2011**, 187103 (2011). 10.1155/2011/187103 PMC3051160 21403873

[7] Lange B, Mueller JK, Leweke FM, Bumb JM (2017). How gender affects the pharmacotherapeutic approach to treating psychosis–a systematic review. Expert Opin. Pharmacother..

[8] Ceskova E, Prikryl R, Libiger J, Svancara J, Jarkovsky J (2015). Gender differences in the treatment of first-episode schizophrenia: results from the European First Episode Schizophrenia Trial. Schizophr. Res..

[9] Seeman MV (2010). Schizophrenia: women bear a disproportionate toll of antipsychotic side effects. J. Am. Psychiatr..

[10] Melkersson, K. I., Hulting, A. L. & Rane, A. J. Dose requirement and prolactin elevation of antipsychotics in male and female patients with schizophrenia or related psychoses. *Br. J. Clin. Pharmacol*. 10.1046/j.1365-2125.2001.01352.x (2001). 10.1046/j.1365-2125.2001.01352.x PMC2014456 11318766

[11] National Institute of Health and Clinical Excellence. Psychosis and schizophrenia in adults. NICE guidelines and treatment management. 10.1002/14651858.CD010823.pub2.Copyright (2014).

[12] Kreyenbuhl J, Buchanan RW, Dickerson FB, Dixon LB (2010). The schizophrenia patient outcomes research team (PORT): Updated treatment recommendations 2009. Schizophr. Bull..

[13] Medicines.org.uk. Summary of product characteristics. Pharmaceutical medicine. http://www.medicines.org.uk/emc/ (2014).

[14] Urichuk L, Prior T, Dursun S, Baker G (2008). Metabolism of atypical antipsychotics: involvement of cytochrome P450 enzymes and relevance for drug-drug interactions. Curr. Drug Metab..

[15] Bergemann N, Kopitz J, Kress KR, Frick A (2004). Plasma amisulpride levels in schizophrenia or schizoaffective disorder. Eur. Neuropsychopharmacol..

[16] Wolbold R (2003). Sex is a major determinant of CYP3A4 expression in human liver. Hepatology.

[17] Bebia Z (2004). Bioequivalence revisited: influence of age and sex on CYP enzymes. Clin. Pharmacol. Ther..

[18] Scandlyn, M. J., Stuart, E. C. & Rosengren, R. J. Sex-specific differences in CYP450 isoforms in humans. *Expert Opin. Drug Metab. Toxicol.*10.1517/17425255.4.4.413 (2008). 10.1517/17425255.4.4.413 18524030

[19] Carrillo JA (2003). Role of the smoking-induced cytochrome P450 (CYP)1A2 and polymorphic CYP2D6 in steady-state concentration of olanzapine. J. Clin. Psychopharmacol..

[20] Levine SZ, Rabinowitz J, Engel R, Etschel E, Leucht S (2008). Extrapolation between measures of symptom severity and change: an examination of the PANSS and CGI. Schizophr. Res..

[21] Kahn, R. S. et al. Amisulpride and olanzapine followed by open-label treatment with clozapine in first-episode schizophrenia and schizophreniform disorder (OPTiMiSE): a three-phase switching study. *Lancet Psychiatry*10.1016/S2215-0366(18)30252-9 (2018). 10.1016/S2215-0366(18)30252-9 30115598

[22] Goldstein JM (2002). Sex differences in clinical response to olanzapine compared with haloperidol. Psychiatry Res..

[23] Usall J, Suarez D, Haro JM (2007). Gender differences in response to antipsychotic treatment in outpatients with schizophrenia. Psychiatry Res..

[24] Jääskeläinen E (2013). A systematic review and meta-analysis of recovery in schizophrenia. Schizophr. Bull..

[25] Boter H (2009). Effectiveness of antipsychotics in first-episode schizophrenia and schizophreniform disorder on response and remission: an open randomized clinical trial (EUFEST). Schizophr. Res..

[26] Fleischhacker WW (2013). Metabolic risk factors in first-episode schizophrenia: baseline prevalence and course analysed from the European First-Episode Schizophrenia Trial. Int. J. Neuropsychopharmacol..

[27] Rettenbacher, M. A. et al. Alterations of glucose metabolism during treatment with clozapine or amisulpride: results from a prospective 16-week study. *J. Psychopharmacol*. 10.1177/0269881106069467 (2007). 10.1177/0269881106069467 17050656

[28] Alosaimi FD (2018). Prevalence and risk factors of hyperprolactinemia among patients with various psychiatric diagnoses and medications. Int. J. Psychiatry Clin. Pract..

[29] Peuskens J, Pani L, Detraux J, De Hert M (2014). The effects of novel and newly approved antipsychotics on serum prolactin levels: a comprehensive review. CNS Drugs.

[30] Riecher-Rössler A (2017). Oestrogens, prolactin, hypothalamic-pituitary-gonadal axis, and schizophrenic psychoses. Lancet Psychiatry.

[31] Johnsen E (2020). Amisulpride, aripiprazole, and olanzapine in patients with schizophrenia-spectrum disorders (BeSt InTro): a pragmatic, rater-blind, semi-randomised trial. Lancet Psychiatry.

[32] WHO Collaborating Centre for Drug Statistics Methodology. International language for drug utilization research. http://www.whocc.no/ (2014).

[33] Kay SR, Fiszbein A, Opler LA (1987). The positive and negative syndrome scale (PANSS) for schizophrenia. Schizophr. Bull..

[34] Lingjærde, O., Ahlfors, U. G., Bech, P., Dencker, S. J. & Elgen, K. The UKU side effect rating scale: a new comprehensive rating scale for psychotropic drugs and a cross-sectional study of side effects in neuroleptic-treated patients. *Acta Psychiatr. Scand*. 10.1111/j.1600-0447.1987.tb10566.x (1987). 10.1111/j.1600-0447.1987.tb10566.x 2887090

