# Supplementary material for: Sex differences in antipsychotic efficacy and side effects in schizophrenia spectrum disorder: results from the BeSt InTro study
Source: NPJ Schizophr. 2021 Aug 18;7:39. doi: 10.1038/s41537-021-00170-3 (PMC8373883; doi:10.1038/s41537-021-00170-3)
Supplement: Supplementary file 1 — Supplementary Information [file 41537_2021_170_MOESM1_ESM.pdf]

SUPPLEMENTARY INFORMATION

TABLE OF CONTENTS

Content	page
Supplementary table 1	2
Supplementary table 2	3
Supplementary table 3	4
Supplementary table 4	5
Supplementary data: R codes	6

Supplementary table 1

PANSS score decrease from baseline at each timepoint

	Amisulpride (n=52)		Aripiprazole (n=51)		Olanzapine (n=41)	
	Men (n=25)	Women (n=27)	Men (n=39)	Women (n=12)	Men (n=29)	Women (n=12)
**After 1 week**						
PANSS total	-16.3 (3.5)	-7.3 (3.4)	-8.6 (2.7)	-6.3 (4.7)	-10.1 (2.8)	-5.5 (4.3)
PANSS positive	-5.6 (1.0)	-3.5 (1.0)	-2.7 (0.9)	-1.4 (1.6)	-2.7 (0.9)	-1.7 (1.4)
PANSS negative	-2.7 (1.2)	-0.55 (1.2)	-2.1 (0.9)	-0.9 (1.7)	-1.4 (1.1)	-1.4 (1.6)
PANSS general	-8.0 (1.9)	-3.2 (1.9)	-3.8 (1.5)	-4.1 (2.7)	-6.1 (1.6)	-2.4 (2.4)
**After 3 weeks**						
PANSS total	-22.2 (3.5)	-16.2 (3.5)	-12.8 (2.7)	-10.1 (5.1)	-19.4 (3.0)	-11.7 (4.7)
PANSS positive	-7.0 (1.1)	-6.7 (1.0)	-4.2 (0.9)	-4.0 (1.7)	-6.5 (1.0)	-2.8 (1.5)
PANSS negative	-3.4 (1.2)	-1.5 (1.2)	-3.0 (0.9)	-0.9 (1.8)	-2.5 (1.1)	-3.4 (1.7)
PANSS general	-10.9 (1.9)	-8.1 (1.9)	-5.7 (1.5)	-5.4 (2.9)	-10.4 (1.7)	-5.5 (2.6)
**After 6 weeks**						
PANSS total	-25.2 (3.6)	-19.2 (3.8)	-14.9 (3.1)	-15.0 (4.9)	-18.0 (3.01)	-17.1 (5.4)
PANSS positive	-9.3 (1.1)	-7.2 (1.1)	-6.8 (1.0)	-4.6 (1.6)	-7.1 (0.98)	-5.6 (1.7)
PANSS negative	-3.5 (1.2)	-1.5 (1.3)	-0.5 (1.1)	-2.7 (1.7)	-0.9 (1.1)	-4.8 (2.0)
PANSS general	-12.5 (2.0)	-10.5 (2.1)	-7.5 (1.8)	-7.8 (2.8)	-10.1 (1.7)	-6.9 (3.0)
**After 12 weeks**						
PANSS total	-25.5 (3.8)	-19.9 (4.0)	-17.9 (3.3)	-21.8 (5.5)	-21.2 (3.1)	-11.2 (5.4)
PANSS positive	-10.2 (1.1)	-7.9 (1.2)	-7.3 (1.1)	-8.8 (1.9)	-8.0 (1.0)	-5.4 (1.7)
PANSS negative	-1.2 (1.3)	-1.7 (1.4)	-1.7 (1.2)	-1.3 (1.9)	-1.7 (1.2)	-1.1 (2.0)
PANSS general	-14.2 (2.1)	-10.3 (2.2)	-8.9 (1.9)	-11.9 (3.1)	-11.6 (1.7)	-4.8 (3.0)
**After 26 weeks**						
PANSS total	-34.4 (4.1)	-18.8 (4.2)	-18.8 (3.7)	-27.7 (5.8)	-18.7 (3.5)	-13.0 (6.3)
PANSS positive	-13.3 (1.2)	-7.5 (1.3)	-7.9 (1.2)	-11.5 (2.0)	-7.8 (1.1)	-6.2 (2.0)
PANSS negative	-4.7 (1.4)	-1.7 (1.4)	-1.1 (1.3)	-2.3 (2.1)	-1.5 (1.3)	-1.2 (2.3)
PANSS general	-16.4 (2.3)	-9.7 (2.3)	-9.7 (2.1)	-14.1 (3.3)	-9.5 (1.9)	-5.8 (3.5)
**After 39 weeks**						
PANSS total	-39.3 (4.1)	-24.5 (4.0)	-20.8 (3.9)	-23.1 (9.1)	-24.7 (3.5)	-14.5 (6.3)
PANSS positive	-13.4 (1.2)	-8.7 (1.2)	-8.5 (1.3)	-8.3 (3.0)	-9.0 (1.1)	-6.7 (2.0)
PANSS negative	-6.3 (1.4)	-2.3 (1.4)	-0.90 (1.4)	-3.0 (3.2)	-2.8 (1.3)	-2.0 (2.3)
PANSS general	-19.7 (2.3)	-13.4 (2.2)	-11.3 (2.2)	-12.1 (5.1)	-13.0 (1.9)	-6.0 (3.5)
**After 52 weeks**						
PANSS total	-38.9 (4.2)	-19.7 (4.2)	-23.2 (4.4)	-24.1 (7.6)	-25.3 (3.3)	-23.2 (6.3)
PANSS positive	-13.3 (1.3)	-7.9 (1.3)	-10.9 (1.5)	-8.2 (2.6)	-8.3 (1.1)	-8.2 (2.0)
PANSS negative	-6.7 (1.4)	-2.3 (1.4)	-1.5 (1.6)	-3.7 (2.7)	-3.7 (1.2)	-6.5 (2.3)
PANSS general	-18.9 (2.3)	-9.5 (2.3)	-10.6 (2.5)	-12.4 (4.3)	-13.3 (1.8)	-8.8 (3.5)

Notes

n=number in the total sample; PANSS=the Positive and Negative Syndrome Scale. Analysis based on linear mixed model with time as a categorical variable. PANSS score decrease at each timepoint is presented with SE.

Supplementary table 2

Comparison between medications for PANSS score reduction

	Men (n=93)				
	Amisulpride (n=25)	Aripiprazole (n=39)	p value[1]	Olanzapine (n=29)	p value[2]
PANSS total	-0.60 (0.07)	-0.35 (0.07)	**0.014***	-0.33 (0.06)	**0.004***
PANSS positive	-0.20 (0.02)	-0.17 (0.02)	0.399	-0.12 (0.02)	**0.007***
PANSS negative	-0.10 (0.02)	-0.00 (0.02)	**<0.001***	-0.05 (0.02)	0.057
PANSS general	-0.29 (0.04)	-0.19 (0.04)	**0.050**	-0.16 (0.03)	**0.011***

	Women (n=51)				
	Amisulpride (n=27)	Aripiprazole (n=12)	p value[1]	Olanzapine (n=12)	p value[2]
PANSS total	-0.30 (0.06)	-0.51 (0.13)	0.118	-0.29 (0.11)	0.956
PANSS positive	-0.11 (0.02)	-0.21 (0.04)	**0.032**	-0.12 (0.04)	0.582
PANSS negative	-0.04 (0.02)	-0.06 (0.04)	0.591	-0.06 (0.04)	0.609
PANSS general	-0.16 (0.03)	-0.26 (0.07)	0.187	-0.11 (0.06)	0.485

Notes n=number in the total sample; PANSS=the Positive and Negative Syndrome Scale. Analysis based on linear mixed model with time as continuous variable. PANSS score decrease per week is presented with SD. Amisulpride was used as reference category. P value[1]=amisulpride vs aripiprazole; p value[2]=amisulpride vs olanzapine. Differences significant with α=0.05 are shown in bold. P values with * remained significant after FDR correction.

Supplementary table 3

Comparison between medications for mean side-effects

	Men (n=93)			
	Amisulpride (n=25)	Aripiprazole (n=39)	Olanzapine (n=29)	p value
Neurologic side-effects (UKU)	0.37 (0.56)	0.46 (0.44)	0.34 (0.47)	0.155
Sexual side-effects (UKU)	0.26 (0.42)	0.34 (0.43)	0.18 (0.35)	**0.006**
BMI increase (kg/m2)	0.90 (1.14)	0.64 (1.28)	1.48 (1.27)	**<0.001**
Glucose level (mmol/L)	5.08 (0.62)	5.71 (1.45)	5.40 (1.76)	**0.001**
Prolactin level (mIU/L)	920.3 (553.0)	265.9 (328.4)	376.5 (223.5)	**<0.001**
Tension in breasts (UKU)	N/A	N/A	N/A	N/A
Decreased menstruation (UKU)	N/A	N/A	N/A	N/A

	Women (n=51)			
	Amisulpride (n=27)	Aripiprazole (n=12)	Olanzapine (n=12)	p value
Neurologic side-effects (UKU)	0.31 (0.38)	0.49 (0.40)	0.38 (0.46)	**0.033**
Sexual side-effects (UKU)	0.37 (0.48)	0.23 (0.44)	0.34 (0.54)	0.183
BMI increase (kg/m2)	1.34 (2.27)	-0.04 (1.46)	0.24 (1.25)	**<0.001**
Glucose level (mmol/L)	5.15 (0.82)	5.47 (1.63)	5.00 (0.68)	0.082
Prolactin level (mIU/L)	1869.2 (1212.5)	307.4 (162.3)	667.7 (542.1)	**<0.001**
Tension in breasts (UKU)	0.17 (0.46)	0.25 (0.70)	0.02 (0.15)	0.078
Decreased menstruation (UKU)	0.65 (1.15)	0.20 (0.66)	0.00 (0.00)	**<0.001**

Notes n=number in the total sample; BMI=Body Mass index; kg=kilograms; mmol=millimole; mIU; milli International Units; L=litre. Analyses based on ANOVA. Data are mean values over all timepoints of the follow-up period with SD. Differences significant with α=0.05 are shown in bold. Post-hoc analyses were performed for significant group differences to evaluate differences between specific medication groups.

Supplementary table 4

Comparison between medications for side-effects post-hoc analyses

	Men (n=93)					
	Amisulpride (n=25)	Aripiprazole (n=39)	Olanzapine (n=29)	P value[1]	P value[2]	P value[3]
Neurologic side-effects (UKU)	0.37 (0.56)	0.46 (0.44)	0.34 (0.47)	Not done	Not done	Not done
Sexual side-effects (UKU)	0.26 (0.42)	0.34 (0.43)	0.18 (0.35)	0.267	0.225	**0.004**
BMI increase (kg/m2)	0.90 (1.14)	0.64 (1.28)	1.48 (1.27)	0.394	**0.014**	**<0.001**
Glucose level (mmol/L)	5.08 (0.62)	5.71 (1.45)	5.40 (1.76)	**<0.001**	0.145	0.173
Prolactin level (mIU/L)	920.3 (553.0)	265.9 (328.4)	376.5 (223.5)	**<0.001**	**<0.001**	**0.031**
Tension in breasts (UKU)	N/A	N/A	N/A			
Decreased menstruation (UKU)	N/A	N/A	N/A			

	Women (n=51)					
	Amisulpride (n=27)	Aripiprazole (n=12)	Olanzapine (n=12)	P value[1]	P value[2]	P value[3]
Neurologic side-effects (UKU)	0.31 (0.38)	0.49 (0.40)	0.38 (0.46)	**0.025**	0.620	0.383
Sexual side-effects (UKU)	0.37 (0.48)	0.23 (0.44)	0.34 (0.54)	Not done	Not done	Not done
BMI increase (kg/m2)	1.34 (2.27)	-0.04 (1.46)	0.24 (1.25)	**<0.001**	**0.041**	0.835
Glucose level (mmol/L)	5.15 (0.82)	5.47 (1.63)	5.00 (0.68)	Not done	Not done	Not done
Prolactin level (mIU/L)	1869.2 (1212.5)	307.4 (162.3)	667.7 (542.1)	**<0.001**	**<0.001**	0.126
Tension in breasts (UKU)	0.17 (0.46)	0.25 (0.70)	0.02 (0.15)	Not done	Not done	Not done
Decreased menstruation (UKU)	0.65 (1.15)	0.20 (0.66)	0.00 (0.00)	**0.017**	**<0.001**	0.598

Notes n=number in the total sample; BMI=Body Mass index; kg=kilograms; mmol=millimole; mIU; milli International Units; L=litre. Analyses based on Tukey's post-hoc tests. Data are mean values over all timepoints of the follow-up period with SD. Differences significant with α=0.05 are shown in bold. p value[1]=amisulpride vs. aripiprazole, p value[2]=amisulpride vs. olanzapine, p value[3]=aripiprazole vs. olanzapine. Not done=post-hoc analysis not done, because no significant differences in ANOVA.

Supplementary data

R codes

```
# Simplified version of syntaxes that have been used in the analyses

# Load required libraries

library(devEMF)

library(dplyr)

library(haven)

library(lsmeans)

library(nlme)

# Commands used for reading and preparing data have been removed

# Modell 1, Comparison between men and women for PANSS score reduction, time as continuous variable

data$time = as.numeric(data$time)

modell1 = lme(panss ~ time + gender + time:gender, random = ~1|Patient_ID, data=data)

# Comparison between men and women for mean side-effects

group_by(data, gender) %>% summarise(mean=mean(side_effect), sd=sd(side_effect))

# Modell 2, Comparison between men and women for PANSS score reduction, time as categorical variable

data$time = as.factor(data$time)

modell2 = lme(panss ~ time + gender + time:gender, random = ~1|Patient_ID, data=data)

# Comparison between medications for mean side-effects

group_by(data, medication) %>% summarise(mean=mean(side_effect), sd=sd(side_effect))

# Commands used for storing tables and figures have been removed
```
