# Supplementary material for: Sex differences in antipsychotic efficacy and side effects in schizophrenia spectrum disorder: results from the BeSt InTro study
Source: NPJ Schizophr. 2021 Aug 18;7:39. doi: 10.1038/s41537-021-00170-3 (PMC8373883; doi:10.1038/s41537-021-00170-3)
Supplement: Supplementary file 2 — Reporting Summary [file 41537_2021_170_MOESM2_ESM.pdf]

nature research

Corresponding author(s): I. E. C. Sommer

Last updated by author(s): Jun 30, 2021

Reporting Summary

Nature Research wishes to improve the reproducibility of the work that we publish. This form provides structure for consistency and transparency in reporting. For further information on Nature Research policies, see our Editorial Policies and the Editorial Policy Checklist.

Statistics

For all statistical analyses, confirm that the following items are present in the figure legend, table legend, main text, or Methods section.

n/a	Confirmed	
☐	☒	The exact sample size (*n*) for each experimental group/condition, given as a discrete number and unit of measurement
☐	☒	A statement on whether measurements were taken from distinct samples or whether the same sample was measured repeatedly
☐	☒	The statistical test(s) used AND whether they are one- or two-sided<br>*Only common tests should be described solely by name; describe more complex techniques in the Methods section.*
☐	☒	A description of all covariates tested
☐	☒	A description of any assumptions or corrections, such as tests of normality and adjustment for multiple comparisons
☐	☒	A full description of the statistical parameters including central tendency (e.g. means) or other basic estimates (e.g. regression coefficient) AND variation (e.g. standard deviation) or associated estimates of uncertainty (e.g. confidence intervals)
☐	☒	For null hypothesis testing, the test statistic (e.g. *F*, *t*, *r*) with confidence intervals, effect sizes, degrees of freedom and *P* value noted<br>*Give P values as exact values whenever suitable.*
☒	☐	For Bayesian analysis, information on the choice of priors and Markov chain Monte Carlo settings
☒	☐	For hierarchical and complex designs, identification of the appropriate level for tests and full reporting of outcomes
☐	☒	Estimates of effect sizes (e.g. Cohen's *d*, Pearson's *r*), indicating how they were calculated

Our web collection on statistics for biologists contains articles on many of the points above.

Software and code

Policy information about availability of computer code

Data collection	Between October 20, 2011, and December 30, 2016, 359 patients were assessed for eligibility in three psychiatric centres in Norway, and one in Austria. A total of 144 patients met the inclusion criteria and was included in the study. Data collection was completed in December 2017.
Data analysis	Data were analyzed with the R project voor statistical computing (www.R-project.org)

For manuscripts utilizing custom algorithms or software that are central to the research but not yet described in published literature, software must be made available to editors and reviewers. We strongly encourage code deposition in a community repository (e.g. GitHub). See the Nature Research guidelines for submitting code & software for further information.

Data

Policy information about availability of data

All manuscripts must include a data availability statement. This statement should provide the following information, where applicable:

- Accession codes, unique identifiers, or web links for publicly available datasets
- A list of figures that have associated raw data
- A description of any restrictions on data availability

According to the Norwegian law, data sharing requires approvals from the Regional Committees for Medical and Health Research Ethics, and from the Data Protection Officer at Haukeland University Hospital. The data are therefore not publicly available. The data that support these findings can be provided by Erik Johnsen, Norwegian Centre for Mental Disorders research at Haukeland Univeristy Hospital, upon reasonable request.

Field-specific reporting

Please select the one below that is the best fit for your research. If you are not sure, read the appropriate sections before making your selection.

☒ Life sciences ☐ Behavioural & social sciences ☐ Ecological, evolutionary & environmental sciences

For a reference copy of the document with all sections, see nature.com/documents/nr-reporting-summary-flat.pdf

Life sciences study design

All studies must disclose on these points even when the disclosure is negative.

Sample size	In the BeSt InTro study, 144 patients were included (93 men and 51 women)
Data exclusions	No data was excluded from the analysis
Replication	NA
Randomization	Patients in the BeSt InTro study were randomized to a sequence of the three antipsychotics (amisulpride, aripiprazole and olanzapine) and preferably the first drug on the list was chosen as study drug. If the first drug could not be used due to contra-indications or prior negative experiences, the next drug on the list was offered.
Blinding	Randomization was open to the treating psychiatrist, the physician and the patient, but all research personnel involved in the study assessments was blinded.

Reporting for specific materials, systems and methods

We require information from authors about some types of materials, experimental systems and methods used in many studies. Here, indicate whether each material, system or method listed is relevant to your study. If you are not sure if a list item applies to your research, read the appropriate section before selecting a response.

Materials & experimental systems

n/a	Involved in the study	
☒	☐	Antibodies
☒	☐	Eukaryotic cell lines
☒	☐	Palaeontology and archaeology
☒	☐	Animals and other organisms
☐	☒	Human research participants
☐	☒	Clinical data
☒	☐	Dual use research of concern

Methods

n/a	Involved in the study	
☒	☐	ChIP-seq
☒	☐	Flow cytometry
☒	☐	MRI-based neuroimaging

Human research participants

Policy information about studies involving human research participants

Population characteristics	144 patients (93 men and 51 women) aged ≥ 18 with a diagnosis within the schizophrenia spectrum according to the ICD-10 diagnoses F20-F29, and with an indication for oral antipsychotic drug therapy were included. Patients were eligible for the study if they had a score of ≥ 4 on at least one of the following items in the Positive and Negative Syndrome Scale: delusions, hallucinatory behavior, grandiosity, suspiciousness/persecution or unusual thought content.
Recruitment	Patients with symptoms of acute psychosis in the schizophrenia spectrum were recruited consecutively from the catchment areas of Helse Bergen and the Medizinische Universität, Innsbruck.
Ethics oversight	The BeSt InTro study was approved in Norway by the Regional Committees for Medical and Health Research Ethics, and by the Norwegian Medicines Agency, and in Austria by the Etikkommission der Medizinische Universität Innsbruck, and the Austrian Federal Office for Safety in Health Care (BASG). Clinical monitoring according to ICH-GCP was done by the Department of Research and Development, Haukeland University Hospital in Norway, and by the Clinical Trial Centre at the Medical University Innsbruck in Austria.

Note that full information on the approval of the study protocol must also be provided in the manuscript.

Clinical data

Policy information about clinical studies

All manuscripts should comply with the ICMJE guidelines for publication of clinical research and a completed CONSORT checklist must be included with all submissions.

Clinical trial registration	This trial is registered with ClinicalTrials.gov, number NCT01446328
Study protocol	The study protocol is included in the supplemetary material of the main paper: Johnsen, E. et al. Amisulpride, aripiprazole, and olanzapine in patients with schizophrenia-spectrum disorders (BeSt InTro): a pragmatic, rater-blind, semi-randomised trial. The Lancet Psychiatry 7, 945–954 (2020).
Data collection	Data were collected in three academic centres of psychiatry in Norway (Bergen, Trondheim, Stavanger), and one in Austria (Innsbruck) between October 20, 2011, and December 30, 2016
Outcomes	Outcomes were sex differences in dose, dose-corrected serum levels, efficacy and tolerability. Doses were analyzed as defined daily doses (DDD), with 1 DDD equaling 400 mg for amisulpride, 15 mg for aripiprazole and 10 mg for olanzapine. Serum levels were measured at each study visit. Antipsychotic efficacy was assessed with the structured clinical interview for the PANSS. The patient rated version of the UKU side-effect rating scale was used to follow the development of side-effects, with 0=no side-effects, 1=mild side-effects that do not interfere with performance, 2=side-effects that interfere moderately with performance and 3=side-effects that interfere markedly with performance. BMI, glucose levels and serum prolactin levels were assessed at each study visit.
